# Communicating medicine: new ways, new audiences

**DOI:** 10.1177/01410768231220316

**Published:** 2024-01-04

**Authors:** Martin McKee

**Affiliations:** Faculty of Public Health and Policy, London School of Hygiene & Tropical Medicine, London WC1H 9SH, UK

## Writing medicine

The coronavirus disease 2019 pandemic was a scientific success but, in many countries, a political failure. Scientists worked round the clock to understand a virus that was spreading rapidly across the world. They decoded its genome and established systems to monitor its evolution. They tracked its spread and developed models to explain and anticipate its transmission. They developed, tested and brought to market innovative mRNA vaccine technologies in record time. They showed that it was possible, in the RECOVERY trial, to go from finalising a clinical trial protocol to recruiting patients in a matter of days. And they created systems to track the responses of the public in real time, using methods such as tracking mobility and symptoms on smartphones.

Collectively, this work generated information on an unprecedented scale. But information is only useful if it is used and for it to be used it must be understood and trusted. This means that it must be communicated to those who should act on it.

There is, of course, a well-established mechanism for doing this. It is through the medium of the peer-reviewed journal. A team of authors writes a paper, usually following the traditional format of an introduction, saying why what they have studied is important, methods, setting out what they did, results, describing what they found, and discussion, saying why it matters. They send it to the editor of a journal who decides whether it is of interest to them and should be sent to reviewers, who should be experts in the relevant field. Unfortunately, the explosion of research means that potential reviewers are overwhelmed with requests, leading to lengthy delays as the editor pursues others, and eventually the reviews are returned. Sometimes they improve the paper but often they do not. And if the paper is published, it may be picked up by the mass media or find its way onto the desk of a politician.

Yet there is now widespread agreement that this system is struggling or, from some perspectives, broken. Many of the concerns raised by a former editor of the *BMJ* in the pages of this journal still apply, to some extent.^1^ It is often slow and, in some cases, glacial. The peer-review process can fail to identify papers with problems or prevent the publication of those that are important. Coverage of the findings can be inaccurate, especially when the paper is accompanied by a press release that is misleading, perhaps because it was drafted by someone seeking to overstate the importance of their findings.^2^ And if it does reach a politician or, more often, their advisers, it may be virtually incomprehensible. And it rarely reaches the public, who in many cases have paid for the research through their taxes or charitable giving, and whose engagement is often essential if it is to lead to change.

We know that the public want to know more about science, and especially medicine. Medical books are often among the best sellers. Television programmes attract very large audiences. The daily briefings by Sir Chris Whitty and his colleagues were viewed by millions. Although not on the same scale, the weekly briefings that we on Independent SAGE provided, where we asked the public about their concerns and tried to respond in ways that were understandable, attracted tens of thousands.

But what this experience has taught us is that communication, especially when it involves complex issues, is an ability that not everyone possesses. Training, for example in media skills, can help, but it is often defensive, designed to reduce the risk of making mistakes. And the mass media are only one of many outlets that can be used to explain what is important.

## The past

Previously, the medical profession was often suspicious of those who spoke directly to the public. In 1934, the General Medical Council (GMC) resolved that if doctors did broadcast, they should do so anonymously, a view restated by the British Medical Association in 1956, concerned about the power of doctors with high status. Its Ethics Committee warned that the public might not realise that ‘Lord X is capable of talking nonsense or that Professor Y has a bee in his bonnet’.^3^

These attitudes acted as a major disincentive to engaging in the popularisation of medicine seen today, with a few doctors becoming media stars. But this did not mean that medical themes were absent from public discourse, even if they have incorporated insights from their medical knowledge in different ways.

Anton Chekhov turned to writing to make ends meet as he earned so little from treating the poor in rural Russia. He said that ‘I realise that I have two professions, not one. Medicine is my lawful wife and literature my mistress. When I grow weary of one, I pass the night with the other. Neither of them suffers because of my infidelity’.^4^ His experiences influence his plays. Dr Astrov’s character, in *Uncle Vanya*, captures the loneliness of a country doctor in tsarist Russia, while introducing the idea of a physician as an advocate for the environment.^5^ His account of his journey to Sakhalin, in Russia’s far east, is replete with horrifying descriptions of death and suffering.

His compatriot Mikhail Bulgakov is most famous for his novels *The Master and Margarita* and *The White Guard*. He gave up medicine after experiencing the trauma of the Russian civil war, but not before giving us a fascinating account of medicine in rural Russia in *A Country Doctor’s Notebook*.^6^

The baby was dipped alternately into cold and hot water. He did not make a sound, his head flopping lifelessly from side to side as though on a thread. Then, suddenly there came a noise somewhere between a squeak and a sigh, followed by the first weak, hoarse, cry. He’s alive … alive … And the baby was alive.

Others left the profession soon after completing their studies, perhaps with good reason. Although John Keats passed the exams of the Court of Apothecaries with ease, his surprised fellow students attributed it to his knowledge of Latin rather than medicine.^7^ Yet he still managed to incorporate many medical themes into his poetry.^8^

Arthur Conan Doyle, when a medical student at Edinburgh, undertook the 19th-century equivalent of an elective on a Greenland whaler, a prelude to his later work as a ship’s surgeon. Like Chekhov, he struggled at first to make money from medicine. Sherlock Holmes was, in part, modelled on his surgical teacher from Edinburgh, Joseph Bell. Holmes’ methods involved the skills of observation and inquiry used in diagnosis. Thus, ‘When you have eliminated all which is impossible, then whatever remains, however improbable, must be the truth’.^9^ And of course, Holmes, was accompanied on his adventures by a physician, Dr John Watson, named after one of his colleagues.

Less well known was his contemporary, Margaret Todd, who drew on her experiences at the Edinburgh School of Medicine for Women to write her debut novel *Mona Maclean, Medical Student*, which explored women’s roles in the medical profession (although she did so under a male pseudonym).^10^ Her *Life of Dr Sophia Jex-Blake* described the fight of women to enter the medical profession in the 19th century.^11^

In the 20th century, the playwright Somerset Maugham recalled how, in undergraduate training ‘You will have to learn many tedious things which you will forget the moment you have passed your final exam, but in anatomy it is better to have learned and lost than never to have learned at all,^12^ although he did later invoke this knowledge, rather superficially, when he wrote ‘I’ll give you my opinion of the human race … Their heart’s in the right place, but their head is a thoroughly inefficient organism’.^13^

Jonathan Miller combined medicine with comedy, writing the satirical revue *Beyond the Fringe*. He soon gave up medicine to direct plays and operas, but in 1978, he presented a BBC series, *The Body in Question*, creating controversy by showing the dissection of a cadaver on television.^14^ Five years later, the television series *States of Mind* used conversations with leading psychologists, anthropologists and sociologists to explain the nature of thought.^15^ He described how he spent ‘a lot of my time trying to draw the attention of actors to the minute and subtle details of human behaviour, which was the sort of thing I was looking at when I was a neurologist’.

Oliver Sacks trained in the UK but soon moved to the United States, working with patients experiencing Encephalitis Lethargica, a condition linked to the 1918 influenza pandemic and a reminder, as another pandemic arose a century later, of the long-term sequelae of viral infection. Although fully aware of their surroundings they were unable to move. His book *Awakenings*, which described the remarkable effect of treatment with L-dopa, was made into an Academy Award-nominated film, starring Robert de Niro and Robin Williams. His writing illustrated the enormous power of medical narrative, exemplified by the case studies that comprise his 1985 book *The Man Who Mistook His Wife for a Hat and Other Clinical Tales*.^16^

## The present

The prohibitions on speaking to the public have, fortunately, long gone. Doctors everywhere are expected to be able to communicate effectively and, in the UK, it is a core element of the GMC’s code of Good Medical Practice.^17^ To those names from the past, we can add a growing number of contemporary and, thankfully, more diverse examples. Jed Mercurio’s television series *Cardiac Arrest* was a brutally honest depiction of hospital life. His main character, Dr Andrew Collin, realises that his training has left him woefully unprepared and is shocked by the uncaring attitude of those who should be looking after him. Adam Kay’s book and also a television series *This Is Going to Hurt* paints a similar picture of the experiences of junior doctors.^18^ Both subsequently left medicine.

Siddhartha Mukherjee, an oncologist and author of Pulitzer Prize-winning *The Emperor of All Maladies: A Biography of Cancer*, represents the struggle against cancer as a battle against an ‘adversary [that] is formless, timeless, and pervasive’ in which there are ‘victories and losses, campaigns upon campaigns, heroes and hubris, survival and resilience—and inevitably, the wounded, the condemned, the forgotten, the dead’.^19^

Atul Gawande, a surgeon who started writing for *The New Yorker* soon after starting his residency, is credited with strengthening Barack Obama’s commitment to healthcare reform. His books address topics such as the fallibility of doctors, the importance of intuition when confronted by a medical ‘mystery’, and the many ethical dilemmas that arise in clinical practice. In 2014, he gave the BBC Reith Lectures.^20^

## New media and new audiences

More recently, doctors have seized opportunities offered by new media. Kate Granger, a geriatrician, was diagnosed in her late 20s with a sarcoma that would eventually kill her. Shocked by being told, in an off-hand way, by a doctor she had not previously met that her cancer had spread, she then launched the #hellomynameis campaign, which has been adopted throughout the NHS.^21^ In her book *The Bright Side*, she reminds us: ‘Sometimes in Medicine there are no answers’.^22^

I have long been fascinated by those who reach out in these ways. In 2023, I had the opportunity, in a series of podcasts, to interview some of them. They had brought medical messages to the public in different ways. I explored what motivated them, what they sought to communicate, and who had inspired them.

They include Rachel Clarke, a palliative care doctor who has challenged the ways we think of life and death in a series of books and, like Dom Pimenta,^23^ has described the reality of the clinical front line during the pandemic.^24^ Saliha Mahmood Ahmed, a gastroenterologist and winner of the television competition *Masterchef*, has found innovative ways to explain the science of food and the gut.^25^ Hannah Barham-Brown, a general practitioner trainee and wheelchair user, uses the Salty Women podcasts to tackle disability, gender and politics.^26^ Ben Goldacre, through his Bad Science column in the Guardian, did much to expose problems in research and its application.^27^ Nick Black has written a novel about medical intrigue in 19th century London^28^ to complement his book of guided walks, which illustrates the city’s medical history.^29^ Guddi Singh, a paediatrician and television presenter, was inspired by Bollywood to introduce dance on to her ward. Ian Williams uses graphic novels, in effect stories told through cartoons, to illustrate life in a Welsh general practice,^30^ while Ian Fussell, an advocate for the environment, has deployed drama and poetry at recent COP conferences.^31^ Alice Roberts, who became fascinated by anatomy during her surgical training, has applied her knowledge to archaeology, tracing the human journey through time.^32^ Phil Hammond, as the resident doctor in the satirical magazine Private Eye, speaks truth to power,^33^ exposing a series of medical scandals including that involving cardiac surgery in Bristol. Trish Greenhalgh, as a member of Independent SAGE, reached tens of thousands each week in public briefings during the pandemic, explaining complex issues in ways that were easily understandable.^34^

Others have used their platforms to speak to political leaders. Jason Leitch, the one dentist in the line-up, advised the Scottish government throughout the pandemic and gave frequent media briefings, including filling the slot that, in other times, covered the Saturday football matches, while Richard Horton, with his platform as editor of *The Lancet*, has the ear of many world leaders. Finally, the series includes a couple who are not clinically qualified but convey powerful messages about the challenges of practising medicine in a deprived community, in effect bringing the social determinants of health to the television screen. They are Stephen McGann, better known as Dr Turner in *Call the Midwife*, and his wife and series screenwriter, Heidi Thomas.^35^

## Takeaway messages

So what can one take away from these conversations? First, compared to the past when doctors who broadcast were likely to be Lords or Professors,^3^ a view that reflected the BBC at the time, those who engage with the public today are far more diverse, on all conventional measures (although my interviewees did include some professors and one Knight). Second, they are communicating via a remarkable range of media, with the more traditional novels, newspaper columns, and radio and television joined by guidebooks, cookery books, podcasts and cartoons. Many also have large followings on Twitter but, presumably, their successors, perhaps to be known as a new specialty of ‘medical influencers’, will be communicating via TikTok or other platforms yet to be created. Third, they are willing to confront issues once regarded as taboo, such as sex and death. Fourth, some have found imaginative ways to employ satire and humour to convey serious medical messages and are willing to speak truth to power.

The examples above mostly involve delivering knowledge whereas it will become increasingly necessary to communicate in ways that actively counter disinformation being disseminated for a variety of motives, unfortunately in some cases by doctors. This will require new knowledge of cognitive biases and techniques,^36^ such as inoculation and pre-bunking, if it is to be successful.^37^

The need to communicate medicine more widely, in ways that are easily understandable to a wide audience, is more important than ever in a world where growing numbers of people in some countries are rejecting basic scientific principles and where attitudes to science are becoming highly polarised politically.^38^ Hopefully, this paper, and the examples it contains, will encourage others to think of ways that they can use their knowledge and skills to engage with the public.

The podcasts can be accessed on the BMA website at https://www.bma.org.uk/inspiring-doctors-podcast or by searching for Inspiring Doctors on all the main podcast platforms. I am grateful to Alex Cauvi, at the BMA, whose outstanding skills enabled her to do magic with the material I and my interviewees provided.

## Declarations

### Guarantor

MM.

### Provenance

Not commissioned; editorial review.

## References

[1] Smith R. Peer review: a flawed process at the heart of science and journals. J R Soc Med 2006; 99: 178–182. 16574968 10.1258/jrsm.99.4.178 PMC1420798

[2] Sumner P Vivian-Griffiths S Boivin J Williams A Venetis CA Davies A , et al. The association between exaggeration in health related science news and academic press releases: retrospective observational study. BMJ 2014; 349: g7015. 25498121 10.1136/bmj.g7015 PMC4262123

[3] Anonymous. Doctors on the air. BMJ 1956; 1: 388. 13284337 PMC1978695

[4] McDonald P. The Oxford Dictionary of Medical Quotations. Oxford: Oxford University Press, 2004.

[5] Chekhov A. Uncle Vanya. London: Bloomsbury Publishing, 2016.

[6] Bulgakov M. A Country Doctor's Notebook. London: Random House, 1995.

[7] Keats J. Ode to a Nightingale (Complete Edition). Prague: e-artnow, 2017.

[8] Roe N. John Keats, medicine, and poetry. The Lancet 2021; 397: 962–963. 10.1016/S0140-6736(21)00450-5 33631129

[9] Conan Doyle A . The Sign of Four. Peterborough: Broadview Press, 2010.

[10] Travers G. Mona Maclean, Medical Student. Edinburgh: William Blackwood and Sons, 1894.

[11] Todd MG. The Life of Sophia Jex-Blake. Glasgow: Glasgow University Press, 1918.

[12] Maugham WS. Of Human Bondage. New York: George H Doran Company, 1915.

[13] Maugham WS. The Summing Up. New York: Random House, 2001.

[14] Miller J. The Body in Question. London: Random House, 2011.

[15] Miller J , ed. States of Mind: Conversations with Psychological Investigators. London: Pantheon Books, 1983.

[16] Sacks O. The Man who Mistook His Wife for a Hat and Other Clinical Tales. London: Gerald Duckworth, 1985.

[17] General Medical Council. Good Medical Practice 2023. See www.gmc-uk.org/ethical-guidance/ethical-guidance-for-doctors/good-medical-practice (last checked 4 August 2023).

[18] Kay A. This Is Going to Hurt. London: Picador, 2018.

[19] Mukherjee S. The Emperor of all Maladies: a Biography of Cancer. New York: Simon and Schuster, 2010.

[20] Gawande A. The future of medicine 2014. See www.bbc.co.uk/programmes/b04bsgqn (last checked 4 August 2023).

[21] Granger K. #hello my name is … 2015. See www.hellomynameis.org.uk/ (last checked 4 August 2023).

[22] Granger K. The Bright Side. London: Null, 2011.

[23] Pimenta D. Duty of Care. London: Welbeck Publishing, 2020.

[24] Clarke R. Breathtaking: The UK’s Human Story of Covid. London: Little Brown, 2021.

[25] Ahmed SM. The Kitchen Prescription. London: Yellow Kite, 2023.

[26] Stevens A Barham-Brown H. Salty Women 2023. See www.amazon.co.uk/Salty-Women/dp/B08JK1RYLN (last checked 4 August 2023).

[27] Goldacre B. Bad Science. London: Fourth Estate, 2009.

[28] Black N. The Honourable Doctor. London: Grosvenor House Publishing, 2022.

[29] Black N. Walking London's Medical History Second Edition: Medical History. London: CRC Press, 2012.

[30] Williams I. Bad Doctor: The Troubled Life and Times of Dr Iwan James. Brighton: Myriad Editions, 2014.

[31] GreenFutures. We Still Have a Chance: 12 Climate Stories for 12 Days of COP27. 2022. See https://greenfutures.exeter.ac.uk/we-still-have-a-chance/ (last checked 4 August 2023).

[32] Roberts A. The Incredible Human Journey. London: Bloomsbury, 2010.

[33] Hammond P. Dr Hammond's Covid Casebook. London: Private Eye, 2021.

[34] McKee M Altmann D Costello A Friston K Haque Z Khunti K , et al. Open science communication: the first year of the UK's Independent Scientific Advisory Group for Emergencies. Health Policy 2022; 126: 234–244. 35140018 10.1016/j.healthpol.2022.01.006 PMC8760632

[35] McGann S. Call the Midwife – A Labour of Love: Celebrating Ten Years of Life, Love and Laughter. London: W & N, 2021.

[36] McKee M Stuckler D. Reflective practice: how the World Bank explored its own biases? Int J Health Policy Manag 2015; 5: 79–82. 26927392 10.15171/ijhpm.2015.216 PMC4737545

[37] Roozenbeek J van der Linden S Goldberg B Rathje S Lewandowsky S. Psychological inoculation improves resilience against misinformation on social media. Sci Adv 2022; 8: eabo6254. 36001675 10.1126/sciadv.abo6254 PMC9401631

[38] Burn-Murdoch J. Tragic fallout from the politicisation of science in the US. *Financial Times.* 2023. See www.ft.com/content/d387d40a-4e8c-4502-8ae9-62d7f1426b93 (last checked 4 August 2023).

